# Global media framing, COVID-19 and the issue of vaccination: An empirical inquisition

**DOI:** 10.34172/hpp.2022.23

**Published:** 2022-08-20

**Authors:** Nelson Okorie

**Affiliations:** School of Media and Communication (SMC), Pan-Atlantic University, Lagos, Nigeria

**Keywords:** Health promotion, Health communication, COVID-19, Health literacy, Pandemic

## Abstract

**Background:** This study examined global media framing on issues of vaccination for COVID-19. The framing and media dependency theories were used to understand the potential influence of global media outlets as suppliers of health messages during pandemics.

**Methods:** Content analysis was used to generate qualitative and quantitative data to answer the research questions. The qualitative data provided rich descriptive data about the themes and types of news frames on issues of vaccination for COVID-19, while the quantitative data provided statistical details about the frequency, viewership level and types of news frames on issues of vaccination for COVID-19.

**Results:** The findings of this study showed that conflict and responsibility frames were the predominant frames used to report issues of vaccination for COVID-19. Also, the findings of this study indicated that vaccine safety was the overriding media theme on vaccination issue for COVID-19.

**Conclusion:** The global media serve as suppliers of health communication in developed and developing countries. This study recommended that the global media can spearhead an information campaign to correct misconceptions, misrepresentations and misinformation on issues of vaccination for COVID-19.

## Introduction

 The advent of COVID-19 outbreak has several negative impacts on human capital development in the international community. World Health Organization (WHO) reported that COVID-19 is currently linked with the increasing cases of death in the global community.^1,2^ Scholars agree that COVID-19 is a serious health issue, which has negative snowball effect on global economies.^3-5^ In the same vein, it was observed that “… the plague of COVID-19 has disrupted the health architecture and economic security of most nations across the globe”.^6^ Thus, COVID-19 is a plaque that has negatively engulfed human existence. Furthermore, the COVID-19 outbreak has negative multiplier effect on systems, sectors and institutions in the global community.

 Notably, several international health organisations such as WHO and Centre for Disease and Control (CDC) have recommended vaccination as a primary health solution to curb the outbreak of the disease. These organizations reported that the spread of the viral disease can be averted when a significant part of the world population has been vaccinated.^2,7^ Hence, vaccines for COVID-19 will mitigate the pandemic as well as promote sustainable health security in developed and developing countries.

 In recent times, there have been several debates and discussions about the safety and potency of vaccines for COVID-19. Some of these studies focused on the rumours associated with the vaccines,^8^ while other reports have focused on the potency and importance of the vaccine.^9,10^ However, there are few empirical studies on global media framing on vaccination issues for COVID-19.

 Against this background, this study examined global media framing on the issue of vaccination for COVID-19. This is essential because there is need to generate qualitative and quantitative data on the different perspectives on the issue of vaccination. The significance and usefulness of this study provides empirical data on the different perspectives of issues affecting vaccination for COVID-19 in developed and developing countries. Furthermore, the novelty of this study is in line with the Sustainable Development Goal 3.4, which emphasize on the importance of health information and literacy for infectious and viral disease across the globe. The aims of this study are to examine the viewership level of global media framing on issues of vaccination for COVID-19, to determine the types of global media framing on issues of vaccination for COVID-19 and to assess the recurring themes of global media reports on issues of vaccination for COVID-19.

###  Theoretical framework 

 This study has its anchorage on the framing and media dependency theories. Importantly, the framing theory is an offshoot of the agenda setting theory, which postulates the power of the media to report a unique perspective of an issue, which has national and global relevance. In addition, the thrust of the framing theory stipulates that the manner in which the media presents issues can influence the way individuals make decisions or choices. In essence, the media creates unique frames about happening in any modern society. Scholars identified four types of news frames, which are: (1) Conflict frame, which is a news frame that emphasize the conflict that exist with individuals, institutions and groups (2) Human interest frame, which is a news frame that promotes the interest of humanity (3) Economic consequence, which is a news frame that emphasize an issue in line with the economic consequences (4) Responsibility frame is a news frame that portray an issue in a manner that it attributes responsibility for the cause or solution of the issue.^11^ This study relates to this study, whereby the media creates unique frames about issues of vaccination for COVID-19 in a manner to influence the behaviours of individuals in a global community.

 The locus of the media dependency theory stipulates that the more individuals depend on the media to satisfy their need, the more important the media will play in people’s lives. In essence, the influence of the media is determined by the individuals’ dependency on the media to satisfy their information needs. Another key assumption of this theory propounds that individuals greatly depend on news and information from the media during periods of crises, war or pandemic. In essence, the media becomes the focal point for health information and literacy on issues of national and global significance. As it relates to this study, the media serve as a primary source of health information about the nature and danger of COVID-19 in high and low resource countries. In addition, the media serves as a veritable platform for debates and discussion on prevention measures to curb the COVID-19 pandemic across the globe. Furthermore, the media have created narratives about vaccines as curative measures for COVID-19.

###  Vaccines and COVID-19 pandemic 

 In the field of public health, COVID-19 is an acronym for ‘coronavirus 2019’.^1,12^ From a medical standpoint, COVID-19 is an illness caused by novel coronavirus, which is a known as severe acute respiratory syndrome coronavirus 2 (SAR-CoV-2). COVID-19 is an important health concern that has negatively affected the global community. This viral disease has led to the deaths of more than two million persons in developed and developing countries. The global pandemic has significant impact in Asia, Europe and North America compared to Africa and Australia.

 Interestingly, there have been several variants of COVID-19, which redefines the harmful nature of the viral disease. Several media and medical reports have indicated that there are several genetic variants of COVID-19 that are circulating across the globe. Several medical reports have indicated B.1.1.7 (alpha), B.1.135 (beta), B.1.617.2 (delta) and P.1 (gamma) are some generic variants of COVID-19 are circulating in developed and developing countries. ^4^ Thus, COVID-19 pandemic has become more intricate due to the prevalence of genetic variants of the virus.

 Conversely, several international organisations such WHO has recommended vaccines as priority solutions to curb COVID-19 across the globe. WHO reported that vaccines are essential health interventions to curb the threat of COVID-19 in the global community.^2^ Furthermore, the vaccines are critical life-savers that promote sustainable health development. In recent times, WHO has approved a number of vaccines to serve as preventive solutions to curb the virology of the disease. For example, Pfizer/BioNTech Comirnaty vaccine, the SII/CoviShield and AstraZeneca/AZD1222 vaccines, Moderna COVID-19 vaccine and Sinopharm COVID-19 vaccine are some of the vaccines approved and recommended by WHO.^12^ Thus, vaccines are crucial health solutions to curb the dreaded nature of COVID-19 across the globe.

 Notably, there have been several empirical studies on COVID-19 vaccines. For example, a study examined COVID-19 vaccine rumours and conspiracies and the need for cognitive inoculations against misinformation. The study emphasized that the prevalence of rumours and conspiracies has made several individuals hesitant to undertake vaccination. The study analysed 631 COVID-19 vaccine stories related with online rumours and conspiracies in Facebook, Twitter, Instagram, Google, YouTube, fact checking sites, newspaper sites and television sites. The study found that more than 90% of these items were online rumours, while less than 10% of these items were conspiracies. The study recommended that community-based engagements and risk communication approaches should be adopted to curb the spread of misinformation about vaccines for COVID-19.^8^

 In the same vein, another study explored the effects of information on COVID-19 vaccines effectiveness. The study maintained that information about COVID-19 has a direct impact on the beliefs and efficacy of vaccines. The study also maintained that the need for post-vaccination protective behaviours has less correlation with the beliefs and efficacy of vaccines. The study also indicated that messages on the benefits and risks of the vaccine has less impact on the intentions for vaccinations.^9^

 In a related study investigated goods reason for vaccination and the potential need for mandatory vaccination. The study maintained that mandatory vaccination can be ethically acceptable on the basis of severe peril to public health and great confidence in the efficacy and safety of the vaccines. The study also maintained that sanctions could be given to individuals who refuse to undertake vaccination, when vaccination has been made compulsory. Some of the sanctions identified are: fine payment, community service engagement and suspension of social benefits.^10^

## Materials and Methods

 The research method adopted for this study was content analysis. This research method requires a systematic and objective measurement of variables to achieve the study objectives.^13^ For this study, the content analysis generated qualitative and quantitative data to answer the research questions. The qualitative data provided rich descriptive data about the themes and types of news frames on issues of vaccination for COVID-19, while the quantitative data provided statistical details about the frequency, viewership level and types of news frames on issues of vaccination for COVID-19. Importantly, this study adopted three approaches of analysing data for content analysis, which are descriptive, interpretative and predictive approaches.

 For the purpose of empirical novelty, three global television networks were selected to achieve the study objectives. The selected global television networks are CNN, BBC and Aljazeera. These global media outlets were selected based on network coverage, popularity and continental influence. Importantly, the timeline for this research was March 2021 to July 2021. The choice of this timeline was predicated on the prevalence of vaccines for COVID-19.

 The sampling technique adopted for this study was purposive sampling. This sampling technique was adopted to select news stories that focused on vaccination for COVID-19. The criteria for selecting the news stories are: (1). News stories by the selected global media outlets (2). News stories about vaccines for COVID-19 (3). News stories with viewership and comments on YouTube. The sample size for this study was 30 news stories of the three selected global media outlet. The breakdown of the sample size is as follows: Two news stories will be selected per month for a global media outlet and multiplied by five months.

 The unit of analysis for this study are: Types of headlines, types of frames, level of viewership and themes of media report. The types of headlines used in this study are direct headlines, indirect headlines, question headlines, command headlines, reason-why headline, how-to headline and testimonial headline. The types of framing are: (a). Human interest frame, responsibility frame, economic consequence frame and conflict frame. The level of viewership was low, which captures less than 100 000 views on YouTube; (b) Medium viewership, which captures 101 000 to 1,000 000 views on YouTube, (c) High viewership, which captures less than 1,000 000 views on YouTube. The themes of the media reports are vaccine safety, vaccine supply, vaccine hesitancy, vaccine misconception and mandatory vaccination.

 For this experiential search, two hypotheses were tested to establish if there was a significant association between the viewership level and types of global media framing on vaccination issues for COVID-19; ifthere was a significant association between the types of global media framing and themes of media report of vaccination issues for COVID-19.Chi-square analysis was used to ascertain the association between the nominal variables. Furthermore, contingency co-efficient measure was used to determine the significance and direction of the association between the variables.

## Results

 This studygenerated quantitative and qualitative data on global media framing on issues of vaccination for COVID-19. The data were described, interpreted and made substantial generalization about global media reports on vaccination in developed and developing countries.

Table 1 depicts that the global media outlets reported issues about vaccine safety, vaccine hesitancy, mandatory vaccination and vaccine supplies. CNN reports focused more on issues of vaccination in the United States compared to other countries, while BBC reports focused on issues of vaccination in the United Kingdom compared to other countries. Only Aljazeera reports focused more on issues of vaccination in different countries.

**Table 1:** Global media reports on vaccination issues for COVID-19

**S/N**	**Story title**	**News source**	**Date**	**Views**	**Comments**
1	Trump supporters say why they won’t take vaccine	CNN	March 20, 2021	633,098	9523
2	CDC releases guidance for people vaccinated against COVID-19	CNN	March 8, 2021	364,559	3177
3	Many Evangelicals say they won’t be vaccinated against the vaccine	CNN	April 15, 2021	178,890	6890
4	The origin of COVID-19 vaccine hesitancy in UD- Dr. Sanjay Gupta	CNN	April 8, 2021	61,961	1658
5	Biden announces that US will share more vaccines globally	CNN	May 17, 2021	159,872	4331
6	Absolutely not true: Dr. Gupta reacts to Fox host’s comment	CNN	May 6, 2021	439,319	5084
7	Nurse is willing to lose job to avoid vaccine, hear why	CNN	June 4, 2021	2,285,469	41,099
8	Tappers reacts to doctor unhinged claims about magnets	CNN	June 10, 2021	360,359	5131
9	Why do vaccinated people need to mark? See Gupta’s answer	CNN	July 27, 2021	219,964	5506
10	Just get the stupid shot- unvaccinated man who got COVID-19 speaks out	CNN	July 22, 2021	1,292,698	
11	Hancock praises the fantastic vaccine programme	BBC	March 17, 2021	188,078	1591
12	EU leaders to discuss boosting vaccine supplies	BBC	March 25, 2021	149,098	763
13	Johnson and Johnson vaccine paused over rare blood clots	BBC	April 14, 2021	174,318	1268
14	New questions over the safety of AstraZeneca vaccine for young adults	BBC	April 7, 2021	159,845	820
15	Should pregnant women get the COVID-19 vaccine?	BBC	May 9, 2021	74,745	1223
16	COVID: Pfizer and AstraZeneca jabs are effective against Indian variant	BBC	May 23, 2021	206,762	1504
17	How the race for COVID-19 vaccine was won	BBC	June 22, 2021	75,948	1529
18	Vaccinating children against COVID-19	BBC	June 12, 2021	52,370	1521
19	No jab, no entry: Night club industry angers against COVID passport plan	BBC	July 20, 2021	109,472	1687
20	Fully jabbed arrivals from France must still quarantine	BBC	July 17, 2021	84,849	910
21	Are some COVID-19 vaccines better than others?	Aljazeera	March 3, 2021	113,458	629
22	What’s the future of the AstraZeneca vaccine?	Aljazeera	March 17, 2021	39,519	186
23	AstraZeneca vaccine: safe or not safe?	Aljazeera	April 8, 2021	290,015	622
24	Should governments rethink their coronavirus vaccine roll outs?	Aljazeera	April 13, 2021	5547	41
25	Why are Africans leery of COVID-19 vaccines?	Aljazeera	May 19, 2021	10,085	157
26	WHO reviews Chinese COVID vaccine	Aljazeera	May 3, 2021	77,765	608
27	UK launches nationwide booster vaccine trials	Aljazeera	June 17, 2021	8186	84
28	White House announces COVID-19 global sharing plan	Aljazeera	June 4, 2021	3898	47
29	Will COVID-19 vaccines divide rich and poor nations?	Aljazeera	July 2, 2021	8942	116
30	Vaccine or jail: Duterte warns as COVID’s Delta surges	Aljazeera	July 22, 2021	35,969	792

Table 1 also shows that there were more views and comments for issues about vaccine hesitancy. For example, CNN reported how a nurse was willing to lose her job to avoid vaccine. The nurse explained that she will only be vaccinated, when there are more safety trials for vaccines. This news story had more than 200 000 views on YouTube.

Table 1. indicated that Aljazeera adopted the frequent use of question headlines to report issues on vaccination. For example, Are some COVID-19 vaccines better than others? What’s the future of the AstraZeneca vaccine? AstraZeneca vaccine: safe or not safe? Interestingly, CNN adopted the frequent use of reason-why headlines to report issues about vaccination. For example, ‘Trump supporters say why they won’t take vaccine’, ‘Nurse is willing to lose job to avoid vaccine, hear why’, ‘Why do vaccinated people need to mark? See Gupta’s answer’. BBC adopted the frequent use of direct headlines

Table 2 shows that CNN had more viewership than BBC and Aljazeera on issues of vaccination for COVID-19. The table also shows that Aljazeera had low viewership than CNN and BBC. Interestingly, BBC had sufficient viewership on issues of vaccination for COVID-19. Hence, it can be deduced that the level of viewership on YouTube was significant for CNN and low for Aljazeera.

**Table 2:** Level of viewership

**Categories **	**CNN**	**BBC**	**Aljazeera**	**Total**
High viewership (> 1000000)	2 (6.7%)			2 (6.7%)
Medium viewership (100000-1000000)	7 (23.3%)	6 (20 %)	2 (6.7%)	15 (50 %)
Low viewership (<100000)	1 (3.3%)	4 (13.3 %)	8 (26.7 %)	13 (43.3 %)
Total				30 (100%)

Table 3 shows that the CNN and Aljazeera utilized the conflict frame compared to other news frame. The conflict frame is a type of news frame that emphasize the conflict of individuals, institutions and groups. For example, CNN reported how Donald Trump’s supporters indicated they would not get vaccinated. CNN also reported how Evangelicals argued that they would not get vaccinated.

**Table 3:** Frame types on vaccination issues

**Categories **	**CNN**	**BBC**	**Aljazeera**	**Total **
Human interest frame	1 (3.3%)	2 (6.7%)	2 (6.7%)	5 (16.7 %)
Responsibility frame	2 (6.7%)	4 (13.3 %)	2 (6.7%)	8 (26.7 %)
Consequence frame	1 (3.3%)	1 (3.3%)	1 (3.3%)	3 (9.9 %)
Conflict frame	6 (20 %)	3 (10 %)	5 (16.7 %)	14 (46.7 %)
Total				30 (100%)

Table 3 also indicates that the responsibility frame is another predominant news frame used by CNN, BBC and Aljazeera. The responsibility frame is a type of news frame that portray an issue in a manner that it attributes responsibility for the cause or solution of the issue. For example, Aljazeera reported that UK launches nationwide booster vaccine trials. This story was framed to attribute the responsibility for vaccination as a priority solution to curb the outbreak in UK. Hence, the recurring type of news frames used by global media outlets were conflict and responsibility frames to report issues of vaccination for COVID-19.

Table 4 shows that predominant theme of vaccination issues for COVID-19 was vaccine safety. The issue of vaccine safety has multiples concerns such as clinical records of vaccine safety, vaccine for children, vaccines for pregnant women and vaccine side-effects on young people.

**Table 4:** Media themes on vaccination issues

**Categories **	**CNN**	**BBC**	**Aljazeera**	**Total **
Vaccine supplies	1 (3.3%)	1 (3.3%)	3 (10 %)	5 (16.7 %)
Vaccine safety	2 (6.7%)	7 (23.3%)	4 (13.3 %)	13 (143.3 %)
Vaccine hesitancy	4 (13.3 %)	0 (0%)	1 (3.3%)	5 (16.6 %)
Vaccine misconception	3 (10 %)	1 (3.3%)	1 (3.3%)	5 (16.6 %)
Mandatory vaccination		1 (3.3%)	1 (3.3%)	2 (6.6 %)
Total				30 (100%)

Table 4 also shows that vaccine supplies, vaccine hesitancy and vaccine misconceptions are relevant themes reported by global media outlets. CNN reported more themes about vaccine hesitancy compared to other global media outlets. Hence, it can be deduced that vaccine safety was the dominant theme of global media outlets about vaccination issues for COVID-19.

Table 5 depictsthat there is an association between the paired variables. The table shows the linear association was significant (*P*<0.001). Hence, the test confirms that there is a significant association between the viewership level and types of global media framing on vaccination issues for COVID-19.

**Table 5:** Chi-square test 1

**Categories **	**Values**	**Df**	**Asymptotic significance (2-sided)**
Pearson chi-square	64.686^a^	12	0.000
**Symmetric measure**		
Nominal by nominal	0.827		0.000
Number of valid cases	30		

Table 6 displaysthat there is an association between the paired variables. The table shows the linear association was significant (*P*<0.005). Therefore, the test confirms that there is a significant association between the types of global media framing and themes of media report of vaccination issues for COVID-19.

**Table 6:** Chi-square test 2

**Categories **	**Values**	**Df**	**Asymptotic significance (2-sided)**
Pearson Chi-Square	22.180^a^	6	0.001
**Symmetric Measure**			
Nominal by Nominal	0.657		0.001
Number of valid cases	30		

## Discussion

 Currently, COVID-19 is linked with the increasing cases of death in many developed and developing countries. Also, the outbreak of COVID-19 has negative multiple effects on the economic, political and socio-cultural systems in the global community. Notably, vaccination has been regarded as the priority health intervention to curb the spread of the viral disease across the globe. This study examined the global media framing on vaccination issues for COVID-19.

 The empirical data generated for this study shows that the global media outlets adopted unique types of news headlines to achieve certain purposes. Aljazeera adopted the frequent use of question headlines to report issues on vaccination. For example, Are some COVID-19 vaccines better than others? What’s the future of the AstraZeneca vaccine? AstraZeneca vaccine: safe or not safe? The purpose of the question news headline is to stimulate curiosity, which will be used to persuade the reader or listener. It can be inferred that the use of question headlines was to persuade the listeners to support or reject an idea. CNN adopted the frequent use of reason-why headlines to report issues about vaccination. For example, ‘Trump supporters say why they won’t take vaccine’, ‘Nurse is willing to lose job to avoid vaccine, hear why’, ‘Why do vaccinated people need to mark? See Gupta’s answer’. The purpose of the reason-why headline is to enlighten and influence behaviours. It can be deduced that the use of reason-why headlines was to correct misconceptions and provide more justifications on issues.

 This data-based study indicated that the level of viewership on YouTube was significant for CNN and low for Aljazeera. This result reveals that CNN has more viewership on YouTube than other global media outlets such as BBC and Aljazeera. The implication of this findings indicates that CNN can be regarded as a global news giant in terms of reach, frequency, polarity and YouTube viewership. The empirical data also displayed the recurring type of news frames used by global media outlets were conflict and responsibility frames. The data reveal that there were different conflicts from news anchors, political supporters, Christian groups and health workers on issues of vaccination for COVID-19. These conflicts were premised on issues of vaccine safety and vaccine hesitancy. The responsibility frames focused on concerns for vaccine supplies, vaccine for children and vaccine for pregnant women. The findings of this study support the tenets of the framing theory, which expounds that the media can create news frames in a manner to present certain issues to individuals in any modern society. The implication of this result is that news frames on conflict and responsibility will enlighten individuals about issues of vaccination.

 This study also identified vaccine safety as the predominant media themes on issues of vaccination for COVID-19. Vaccine safety is a public health concern in the global community. This result affirms the positions of scholars; they maintained that messages on the benefits and risks of the vaccine has less impact on the intentions for vaccinations. It can be inferred that the safety and benefits of vaccines will curb hesitancy, misconceptions and public distrust. Furthermore, the safety of vaccines is a major indicator for the downturn of the pandemic across the globe.^5^

 For the quantitative aspect of this study, two hypotheses were tested used chi-square and contingency coefficient measurements. The first hypothesis confirmed that there is a significant association between the viewership level and types of global media framing on vaccination issues for COVID-19. The hypothesis test shows that the strength and direction of the association was significant. The result implies that the level of viewership has a direct connection with the types of news frames. The second hypothesis confirmed that there is a significant association between the types of global media framing and themes of media report of vaccination issues for COVID-19. This result implies that the types of news frame have a strong and positive link with the media themes of vaccination issues. A key contribution of this study is that news frames can used to educate, enlighten and influence behaviours during pandemics.

 Importantly, the major limitation of this study was the inability to use a software to code and analyse the qualitative data. Nevertheless, the qualitative data were manually coded and analysed based on specific parameters used in content analysis.

## Conclusion

 The novelty of this research investigation provides empirical data on global media framing and the different perspectives of issues affecting vaccination for COVID-19 in developed and developing countries. The global media serve as suppliers of health communication in developed and developing countries. The activities of global news outlets can reshape the debate and discussion of perennial issues such as vaccination for COVID-19. Based on the tenets of the media dependency theory, the global community rely on the media for news and information to survive a pandemic. This study recommends that media messages on issues of vaccination should be framed to influence priority and protective behaviours to curb the spread of the virus. Furthermore, the global media can spearhead an information campaign to correct misconceptions, misrepresentations and misinformation on issues of vaccination for COVID-19.

## Authors’ contributions

 NK is the sole contributor of this paper, who is responsible for the problem conceptualisation, research design, data collection, and data analysis.

## Funding

 There was no funding for this research

## Ethical approval

 This was not applicable for this study because all data used in this study have already been published, additional approval from the ethical committee was not applicable.

## Competing interests

 The author declares no potential conflict of interest.

## Disclaimer

 The author claims that this manuscript has not been published in any journal and no part of this paper is copied from other sources.
